# Sunscreen use optimized by two consecutive applications

**DOI:** 10.1371/journal.pone.0193916

**Published:** 2018-03-28

**Authors:** Ida M. Heerfordt, Linnea R. Torsnes, Peter A. Philipsen, Hans Christian Wulf

**Affiliations:** Department of Dermatology, Bispebjerg Hospital, University of Copenhagen, NV Copenhagen, Denmark; Fox Chase Cancer Center, UNITED STATES

## Abstract

Sunscreen users are often inadequately protected and become sunburned. This study aimed to investigate how much two consecutive sunscreen applications increased the quantity of sunscreen applied and decreased the skin area left without sunscreen (missed area) compared to a single application. Thirty-one healthy volunteers wearing swimwear were included and applied sunscreen two consecutive times in a laboratory environment. Participants had pictures taken in black light before and after each application. As sunscreens absorb black light, the darkness of the skin increased with increasing amounts of sunscreen applied. We conducted a standard curve establishing a link between change in picture darkness and quantity of sunscreen. The quantity of sunscreen at selected skin sites as well as the percentage of missed area was determined after each application. Participants had missed a median of 20% of their available body surface after a single application. After double application they had missed 9%. The decrease in missed areas was significant for the whole body surface and for each of the body regions separately. The median participant had applied between 13% and 100% more sunscreen at the selected skin sites after double application than after single application. We recommend double application, especially before intense sun exposure.

## Introduction

To avoid sunburn and skin cancer it is recommended to protect the skin from solar ultraviolet radiation (UVR) by seeking shade, avoiding sun exposure around noon, wearing clothes, and applying sunscreen [1,2]. Sunscreen application is a commonly followed recommendation [3]. Studies have documented that sunscreen users often gain insufficient protection and get sunburned after a single application, especially on holidays in sunny locations [4,5]. To protect against UVR it is important to apply a sufficient quantity of sunscreen on all exposed body sites and not to miss any areas [2,6,7]. In order to accomplish this, sunscreen application before sun exposure and reapplication every second hour during exposure has been recommended by the World Health Organization (WHO) [7,8].

Sunscreen protection is fairly stable over time and 8 hours after sunscreen application about half of the received photoprotection is maintained in spite of bathing and physical activity [9,10]. Consequently it would be an advantage to apply the total daily amount of sunscreen only once before sun exposure. The protection will only slowly decrease during the day and the skin will not be overexposed to UVR before reapplication. We propose that sunscreen should be applied twice consecutively before sun exposure. To the best of our knowledge only two studies have previously investigated the effect of consecutive self-applications on the face or forearm, and no study has investigated the benefit of full body reapplication before sun exposure [8;11].

In connection with another study [3] we asked 117 visitors leaving beaches in Denmark if they had used sunscreen that day and if they had applied sunscreen once or more than once. Fifty percent reported to have used sunscreen on that particular day. Half of the sunscreen users had applied sunscreen once whereas the other half had applied sunscreen more than once. This suggests a general motivation for two consecutive sunscreen applications.

Studies have estimated quantities of sunscreen applied and the percentage of the skin left without sunscreen by use of sunscreens containing fluorescent dyes and measurements of the fluorescence intensity [12–15]. Sunscreens absorb black light, and as a result skin covered with sunscreen appear darker in black light than skin not covered with sunscreen [16]. In the present study we investigate if the ability of sunscreen to absorb black light can be used to determine quantities of sunscreen applied at specific skin sites as well as to quantify skin areas left without sunscreen (missed areas). Our study aimed to use these objective measures to investigate if consecutive double application of sunscreen before sun exposure provides significantly better sunscreen cover than a single application.

## Methods

### Participants

**Fig 1:**
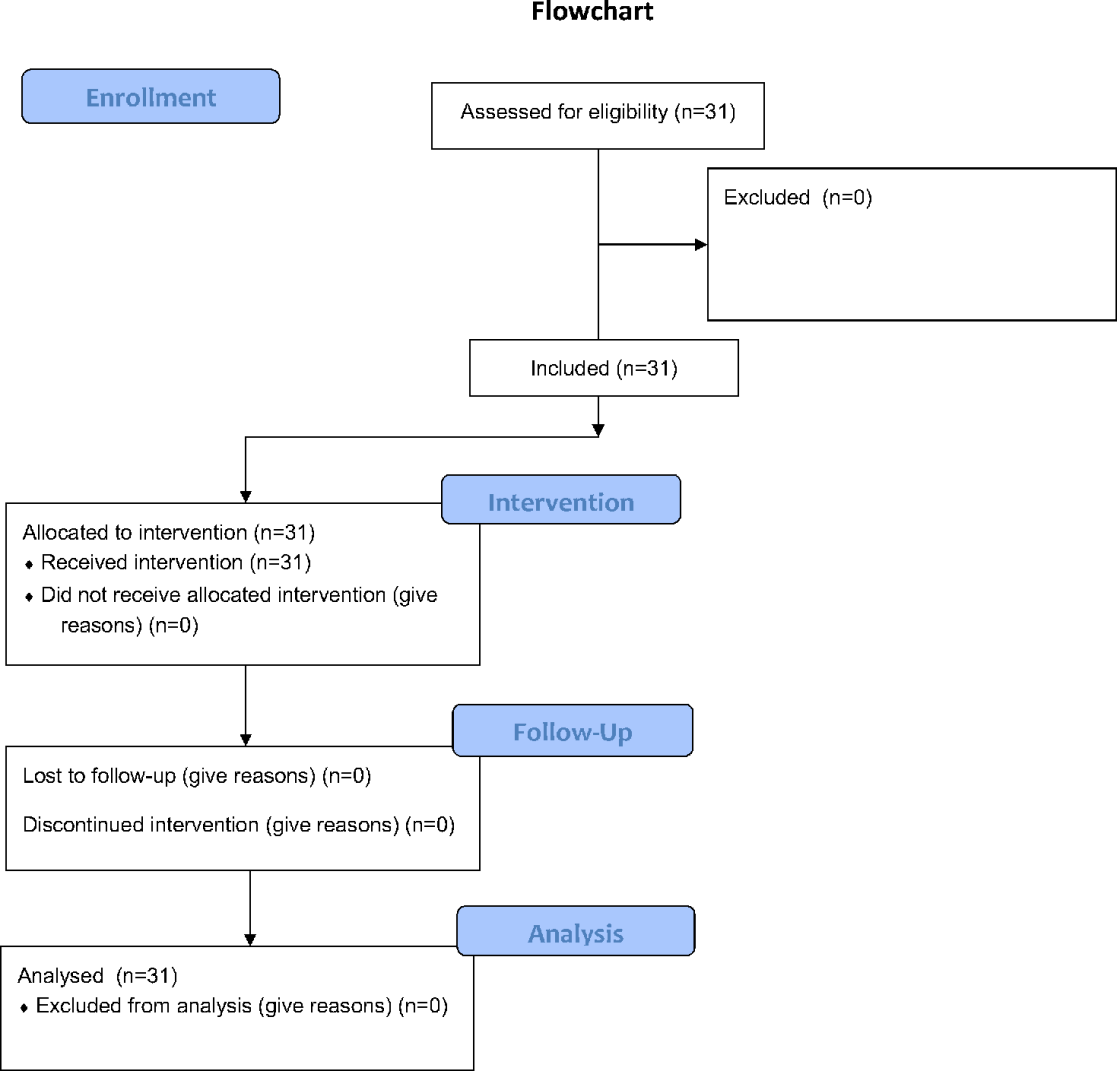
CONSORT participant flow diagram.

Participants were enrolled from a Danish website for recruitment of participants for research projects (www.forsoegsperson.dk). Volunteers were eligible if they were healthy, of European descent, and 18 years of age or older (Fig 1). Volunteers were ineligible if they were suffering from a skin disease, were allergic to the content in sunscreen, were pregnant, or breastfeeding. Participants reported their height and weight. During the whole session women wore a bikini, men wore swimming trunks, and all had their hair pinned up so it did not cover the ears or neck. The majority of beachgoers wear swimwear on Danish beaches during the summer [17]. The regional scientific ethical committee of the Capital Region of Denmark assessed the study protocol in 2014 (H-1-2014-094) and the study was carried out in accordance with this protocol. The committee concluded that the black light exposure was safe and as the intervention in the study consisted only in application of normal sunscreen, no ethical approval was needed. All participants provided informed written consent and completed the study at Bispebjerg Hospital, Denmark, in February 2015. We have previously published results based on these participants regarding the relation between time spent on sunscreen application and achieved photoprotection [18].

Because the local rules do not demand registration, we did not register the trial at clinicaltrials.gov until January 20, 2017 (clinical trial registration number: NCT03033654). The authors confirm that all ongoing and related trials for the intervention are registered.

### Pictures in black light

Before and after each sunscreen application participants had a series of standardized pictures of the whole body taken at fixed distances. We used a digital camera (Canon EOS 450D, EF-S 18–55 mm, Canon Inc., Japan) with an aperture of F:5.6, ISO of 1600 and shutter speed of 1/15. The participants were photographed in a black box with full-height fluorescent tubes (TL08, Philips, The Netherlands) emitting primarily UV-A radiation but also some visible light up to a wavelength of 410 nm. The pictures were assessed as blue channel pictures using GIMP version 2.8.14 (www.gimp.org).

### Sunscreen application

The sunscreen used for testing was Actinica, SPF 50+ (Galderma, Switzerland) [19]. Six squares of 30 cm^2^ each were drawn with a black marker on each participant’s back. Sunscreen was weighed and applied to the squares in as even a layer as possible giving the following quantities in the individual squares; 0 mg/cm^2^; 0.25 mg/cm^2^; 0.5 mg/cm^2^; 1.0 mg/cm^2^; 1.5 mg/cm^2^; and 2.0 mg/cm^2^. Pictures were taken of the squares in black light and the darkness of the pictures depended on reflected light and autofluorescence from the skin, both of which decreased exponentially with increasing quantities of sunscreen.

**Fig 2:**
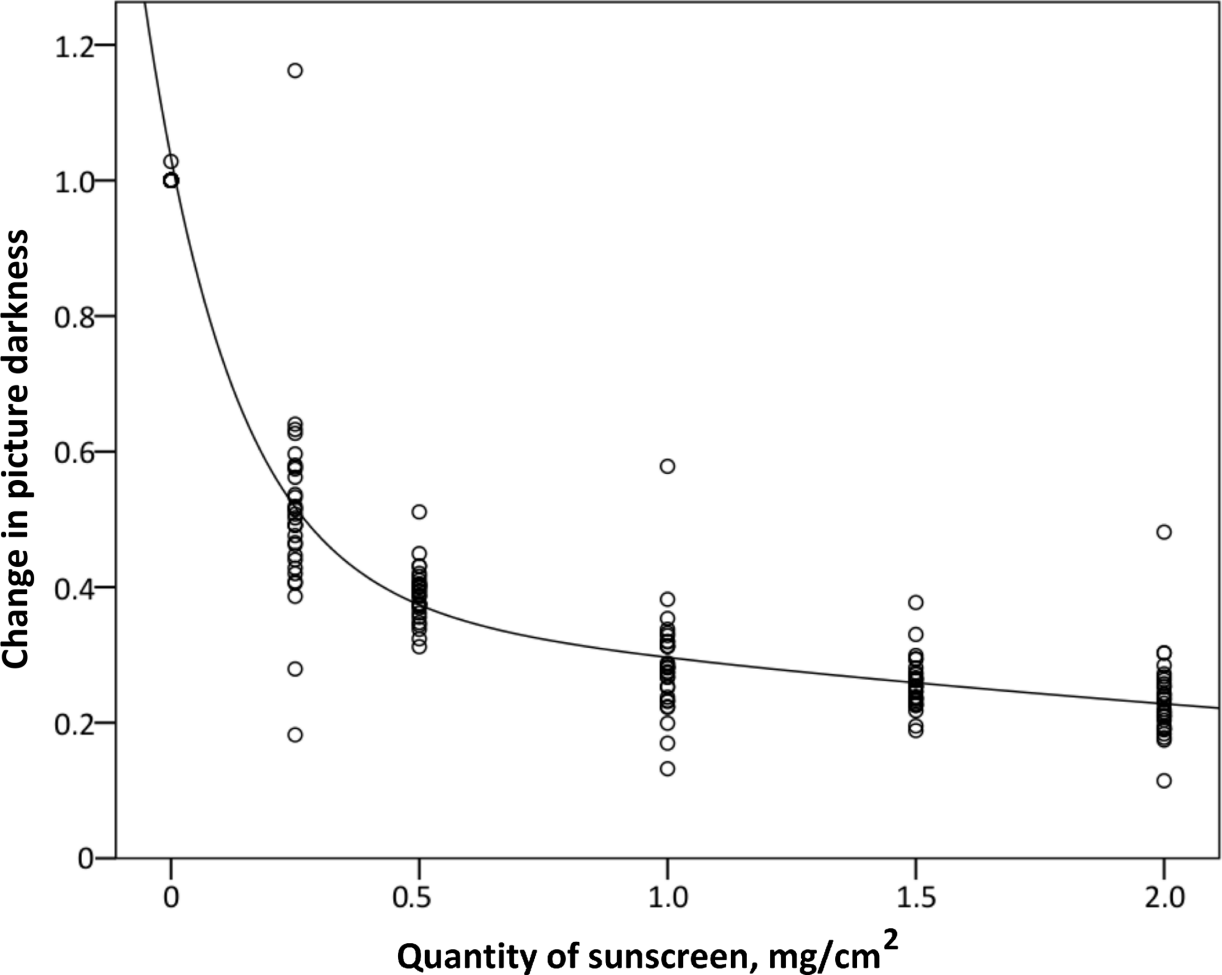
Quantity of sunscreen and picture darkness. The standard curve between quantity of sunscreen, *Q*, and change in darkness of pictures taken in black light, *D*, with the equation: *D* = 0.379×2^−0.367·*Q*^+0.654×2^−8.051·*Q*^.

A standard curve (Fig 2) established the relation between change in picture darkness (*D*) and specific quantity of sunscreen in mg/cm^2^ (*Q*) using nonlinear regression with the following expression: *D* = 0.379×2^−0.367·*Q*^+0.654×2^−8.051·*Q*^. Change in picture darkness was defined as the ratio between picture darkness before application and picture darkness after application.

After the standard curve had been established, participants were asked to apply the provided sunscreen, Actinica, the way they would normally do on a sunny day at the beach in Denmark without taking notice of the squares on their lower back. The quantity of sunscreen on the lower back was not measured after self-application. Participants were asked to perform a second application 20 minutes after the end of the first. No other information or advice was given. To avoid interference with the results, the sunscreen container was weighed before and after each application without the participants’ knowledge. Pictures were taken in black light before and after each sunscreen application.

### Missed areas

A missed area is an accessible skin area left without sunscreen. We divided the body surface accessible for sunscreen application into 11 regions: face; ears; front of neck; back of neck; arms; back of hands; front of trunk; back of trunk; thighs; lower legs, and instep. The percentages of skin left without sunscreen, missed areas (lighter areas on the digital pictures), after single and double application were calculated for each region as the non-covered area divided by the whole skin area. The total body surface area was estimated by self-reported weight and height using Mostellers formula [20]. For each participant we calculated the total missed skin area by adding up the missed areas in each region. The surface area of each region was calculated using the model used in Augustsson *et al*. [21]. The regions contributed: face = 3.5%; front of neck = 1.5%; back of neck = 1%; arms = 14%; hands = 3%; front of trunk, women = 11%; front of trunk, men = 11.5%; back of trunk, women = 11.5%; back of trunk, men = 13%; thighs, women = 21%; thighs, men = 16%; lower legs = 14%, and instep = 3.5%. The soles of the feet, the area of scalp hair, and the skin areas covered with swimwear were not accessible for sunscreen application and are not included in the calculations.

### Quantities of sunscreen

We used two different methods to estimate quantities of sunscreen. The overall quantities of sunscreen were calculated from the weighed amount of sunscreen used divided by the skin area. The actual skin area with sunscreen applied was calculated by subtracting the missed areas, the area covered by swimwear, and the area of the scalp and soles from the total body surface. The overall quantity of sunscreen used on that area was calculated after each application.

Quantities of sunscreen at seven specific skin sites were estimated using the standard curve (Fig 2). The seven skin sites of about 30 cm^2^ each were: forehead; shoulder; chest; upper back; belly; back of thigh, and back of lower leg. Changes in picture darkness was determined after single and double application and converted into the equivalent quantities of sunscreen using the standard curve.

### Statistics

Sample size requirement was based on a study comparing quantity of sunscreen after single and double application [8]. Quantity of sunscreen was estimated to be 1.27 mg/cm^2^ after single application with a standard deviation (SD) of 0.47 mg/cm^2^ and 2.01mg/cm^2^ with an SD of 0.66 mg/cm^2^ after double application. To allow detection of the paired difference in quantity after single and double application with a power of 80% and a significance level of 5% we needed 10 participants. As men and women might use sunscreen differently and to accommodate possible dropouts we chose to include at least 15 men and 15 women [22,23].

The statistical analysis was performed in IBM SPSS statistics version 22 (IBM, Armonk, NY, USA). Since data were not normally distributed the Mann-Whitney test was used to test unpaired data whereas paired data were compared using the Wilcoxon matched-pairs signed-rank test. For correlations Spearman's rank-order correlation was used. P-values less than 0.05 were considered significant. All tests were two-sided.

## Results

**Fig 3:**
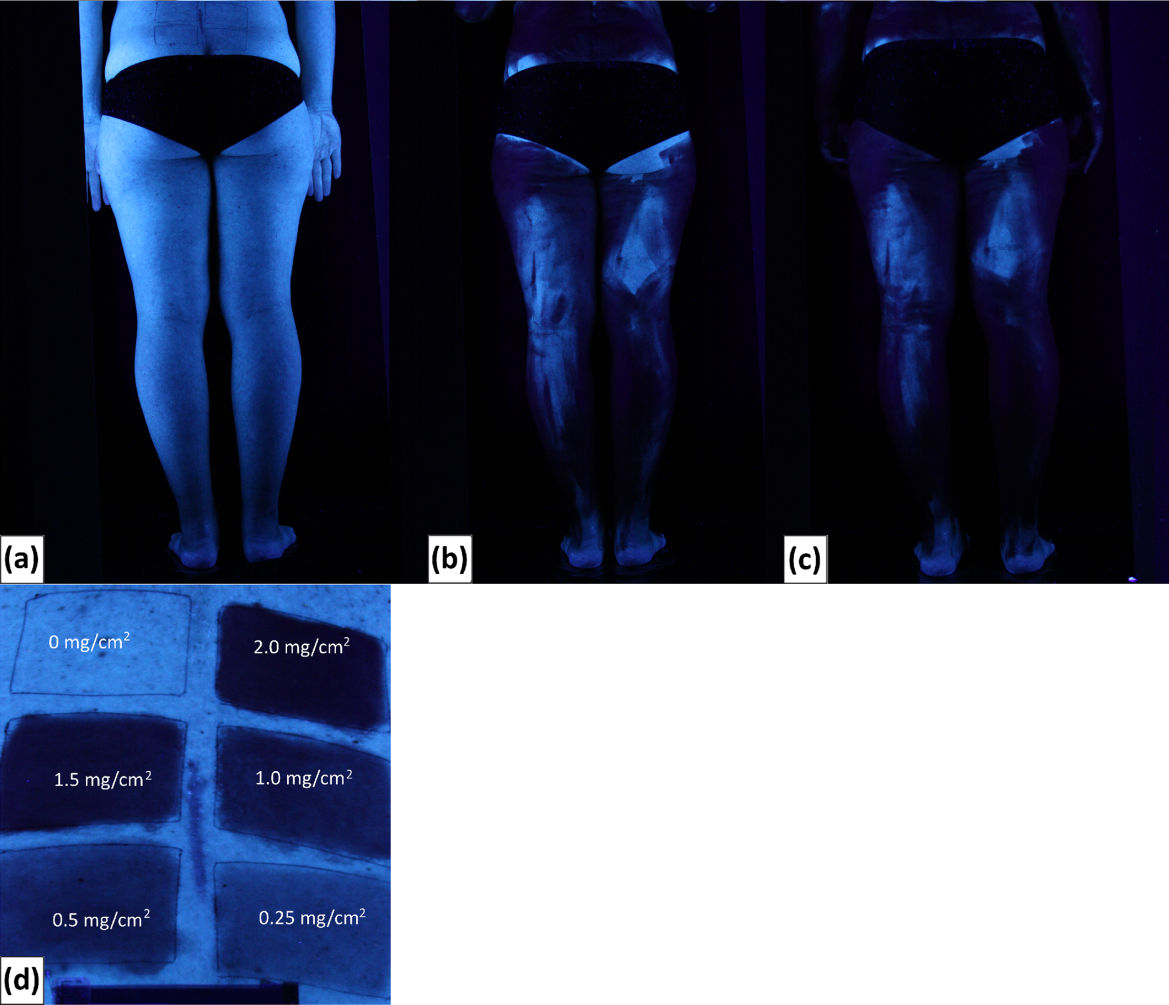
Pictures in black light. Pictures in black light of the back of the legs of a participant before application (a), after single application (b), and after double application (c). Pictures in black light of 6 squares on the lower back of a volunteer with increasing quantities of sunscreen; 0 mg/cm^2^; 0.25 mg/cm^2^; 0.5 mg/cm^2^; 1.0 mg/cm^2^; 1.5 mg/cm^2^; and 2.0 mg/cm^2^. Applied quantities are written on the picture (d). Skin covered with sunscreen appears darker than non-covered skin.

Thirty-one participants, 15 women and 16 men with ages ranging from 19 to 40, were included in the study (Fig 1). Wearing swimwear participants performed two consecutive sunscreen applications in a laboratory environment and had pictures taken in black light before and after each application. See examples of pictures taken in black light in Fig 3.

Participants had a median skin surface accessible for sunscreen application of 1.51 m^2^ (interquartile range (IQR), 1.43 to 1.59 m^2^).

### Missed areas

After single application the participants had covered a median of 1.25 m^2^ (IQR, 0.97 to 1.38 m^2^) skin with sunscreen corresponding to 80% of their accessible skin. After double application they had covered a median of 1.33 m^2^ (IQR, 1.07 to 1.47 m^2^), 91% of their accessible skin. The median absolute decrease in missed area was 6% (IQR, 4 to 10%). The missed areas in different body regions (face; ears; front of neck; back of neck; arms; back of hands; front of trunk; back of trunk; thighs; lower legs, and instep) after single and double application are presented in Table 1. The proportion of participants with many missed areas was greatly reduced after double application compared to after a single application. The decrease was significant both for the whole body surface and for each of the body regions separately (*p* < 0.0004).

**Table 1:** Missed areas.

Body region	Missed area (%) after single application, median (IQR)	Missed area (%) after double application, median (IQR)
Face	3 (1–15)	1 (1–5)
Ears, women	80 (30–100)	44 (18–91)
Ears, men	8 (2–31)	4 (1–17)
Neck, front	6 (1–30)	2 (0–15)
Neck, back	4 (0–11)	1 (0–3)
Trunk, front	7 (2–20)	1 (0–8)
Trunk, back	26 (17–39)	15 (8–32)
Arms	25 (6–36)	4 (1–27)
Hands, back	8 (3–29)	1 (0–3)
Thighs	22 (6–56)	7 (1–48)
Lower leg	5 (1–50)	1 (0–34)
Instep	24 (10–100)	10 (5–55)
**Total**	**20 (10–34)**	**9 (5–25)**

Median percentage of body surface left without sunscreen, missed area, after single and after double application. The decrease in missed area was significant both for the total body surface and for each of the body regions separately (*p* < 0.0004). Abbreviation: IQR = interquartile range.

Men had covered about 10 times more of the skin on the ears than women after both single (*p* = 0.001) and double application (*p* = 0.001), see Table 1. Men and women had the same percentages of missed areas on all the other body regions after both single and double application (*p* > 0.1).

Body regions with the best coverage were the face, the neck, and the lower leg with median missed areas of less than 6% after single application and less than 2% after double application. Regions with the poorest coverage were the ears, the back, the thighs, and the instep with median missed areas of more than 22% after single application and more than 7% after double application.

### Quantities of sunscreen

Participants used a median of 9.0 grams (IQR, 5 to 14 g) of sunscreen at the single application and had used a total of 16.4 grams (IQR, 10 to 27 g) after double application. Applied evenly on the area accessible for sunscreen application this corresponded to a median quantity of sunscreen of 0.60 mg/cm^2^ after single and 1.10 mg/cm^2^ after double application. The amount of sunscreen distributed evenly over the area actually covered by sunscreen corresponded to a median quantity 0.83 mg/cm^2^ (IQR, 0.61 to 1.03 mg/cm^2^) after single application and a total of 1.37 mg/cm^2^ (IQR, 1.01 to 1.87 mg/cm^2^) after double application. The proportion of participants with high quantities of sunscreen applied was greatly increased after double application compared to after a single application.

Quantities of sunscreen on 7 specific skin sites (forehead; shoulder; chest; upper back; belly; back of thigh, and back of lower leg) were determined from the digital pictures using the standard curve (Fig 2 and Table 2). We found no difference in quantity between men and women on any of the skin sites (*p* > 0.07).

**Table 2:** Quantities of sunscreen.

Skin site	Single application, median (IQR), mg/cm^2^	Double application, median (IQR), mg/cm^2^
Shoulder	1.38 (0.48–1.98)	1.74 (1.13–2.36)
Forehead	1.12 (0.71–2.08)	2.21 (1.25–2.94)
Chest	1.05 (0.65–2.06)	2.13 (1.17–2.63)
Belly	0.49 (0.23–1.69)	1.53 (0.39–2.70)
Lower leg, back	0.38 (0.08–0.61)	0.54 (0.11–1.76)
Thigh, back	0.22 (0.06–0.84)	0.49 (0.11–2.52)
Upper back	0.06 (0.01–0.44)	0.18 (0.02–0.55)

Median quantity of sunscreen at specific skin sites after single and double applications, *n* = 31. The increase in quantity of sunscreen from single to double application was significant for all skin sites (*p* < 0.0496) except for the upper back (*p* = 0.19). Abbreviation: IQR = interquartile range.

After single application the shoulders, the forehead and the chest had received the highest quantities of sunscreen (1.38 mg/cm^2^, 1.12 mg/cm^2^ and 1.05 mg/cm^2^) whereas the upper back, the back of the thigh, and the back of the lower leg (0.06 mg/cm^2^; 0.22 mg/cm^2^ and 0.38 mg/cm^2^) had received the lowest quantities. The thickest and the thinnest quantities were found at the same skin sites after both single and double application. On all skin sites a higher quantity had been applied after double than after single application with a median increase of between 13% and 100% depending on the site. The increase was significant at all skin sites (*p* < 0.05) with exception of the upper back (*p* = 0.2).

The quartile of participants (*n* = 8) who used least sunscreen had applied a 22–500% higher quantity of sunscreen on the 7 skin sites after double application than after single application.

### Validation of picture analysis

From the pictures taken in black light we calculated the amount of sunscreen that must have been used for the whole body. This was done by multiplying the skin area covered with sunscreen by the quantity of sunscreen applied in each of the 11 regions. In a few regions (neck, hands, and instep) we did not measure quantity of sunscreen; here we used the quantity measured in the nearest region. The quantity of sunscreen was assumed to be the same all over each region. Addition of these amounts enabled us to estimate the amount of sunscreen used on the whole body after single and double application. From the pictures taken in black light we estimated that participants used a median of 8.1 g of sunscreen at a single application. This was close to the median 9.0 g of sunscreen determined by weighing the sunscreen container before and after application. The median difference between the results from the two methods was only 0.2 g (IQR, -2.9 to 2.5 g). After double application participants had used a median of 14.8 g according to the pictures taken in black light and 16.4 g as determined by weighing, with a median difference of 0.2 g (IQR, -4.2 to 4.2 g). Thus, the results of the two methods were strongly correlated (r = 0.79; *p* < 0.0001). This confirmed that analysis of pictures taken in black light can be used as a method for the determination of quantity of sunscreen applied and area left without sunscreen.

## Discussion

Our study confirmed the hypothesis that double application of sunscreen before sun exposure optimizes sunscreen use compared to a single application. Through analysis of pictures taken in black light we have demonstrated how double application significantly reduced the area of skin left without sunscreen and increased the quantity of sunscreen applied. Causes of inadequate sun protection have been found to be a very uneven application of sunscreen after a single self-application with sunscreen being applied in too thin a layer as well as skin areas being left completely without sunscreen [6,7,24]. Double application proved to address both of these two common problems. Analysis of the quartile of participants who used least sunscreen shows that the intervention also had an effect on this group. However, the problem of self-application of sunscreen on the back of the torso, which is difficult for anatomical reasons, was not solved by double application. Currently the only solution for this problem is help from another person. Our participants were from 19 to 40 years old. It is likely that older participants will have even more difficulty accessing the back of their torsos. Women protected their ears less than men in our study perhaps because women often have their ears covered with hair.

The study found that double application reduced missed areas significantly compared to single application. We find it advisable to apply sunscreen twice before sun exposure to reduce the risk of overexposing missed areas before reapplication. Double application also significantly increases the amount of sunscreen applied and this extra quantity is required to protect against sunburn during sun exposure at locations with high UV index. However, sunscreen is not applied uniformly (see Table 2) and this alone could be a reason to recommend double application even before less intense exposures in order to protect the most poorly covered areas. In addition to avoid sunburn it is advisable to reduce the total lifetime UVR dosage [2]. In this context double application before sun exposure will always be advantageous.

To be protected by an effective SPF, equal to the SPF labeled on the sunscreen container, a sunscreen quantity of 2 mg/cm^2^ must be applied [2;25]. Only 19% of the participants had applied 2 mg/cm^2^ or more sunscreen after double application. After a single application none of them had done so. Studies have shown that the effective SPF is most likely related exponentially to the quantity of sunscreen applied [6, 26]. Accepting this exponentially relation the amount of sunscreen applied thus have given an effective SPF of only 3.2 after the first application. Even though the sunscreen used had and SPF label of 50. After the second application the average quantity was 1.10 mg/cm^2^ and may have given an effective SPF of 8.6.

The results of this study are consistent with the results of the 4 previous studies that have investigated the effect of more than a single application [8, 11, 27, 28]. Two of the studies investigated the change in risk of sunburn by reapplication of sunscreen by an investigator during UVR exposure in addition to a single application before exposure as recommended by WHO. Both found a reduced risk [27, 28]. Only two previous studies have investigated the consequence of two consecutive applications of sunscreen before exposure by weighing the sunscreen container before and after application to the face or forearm [8, 11]. They found double application to increase the quantity of sunscreen applied to nearly 2 mg/cm^2^, which corresponds to quantities we have found on the same body sites.

In our study, we have validated that analysis of pictures taken in black light can be used as a method for determining of quantity of sunscreen applied and area left without sunscreen. This method builds on the ability of sunscreen to absorb black light [29] and a standard curve estimating quantity of sunscreen based on picture darkness. The procedure requires only simple and inexpensive equipment, but must be carried out in a dark room illuminated by black light only. The standard curve shows the best sensitivity in quantities of sunscreen ranging from 0 to 1.0 mg/cm^2^. Fortunately these quantities are those used in real-life settings [3].

Retrospectively, it has appeared that it would have been preferable to use a sunscreen with a lower SPF in our study, as the standard curve would have ended less flat for higher quantities of sunscreen providing more accurate results. Another limitation of the method might be that different SPF and active ingredients may induce varying absorption spectra [29]. This will make it necessary to establish new standard curves for each sunscreen.

In conclusion, our study validated the very simple recommendation that sunscreen should be applied twice before sun exposure, especially before an intense exposure. This recommendation has the advantage that, as opposed to rules about how much sunscreen should be used and how it should be distributed, it does not require any education of the population. The conclusion can be printed in a few words on sunscreen containers: “Apply twice”.

## Supporting information

S1 File Log files of the statistical analyses from IBM SPSS statistics. (DOCX)

S2 File Clinical trial protocol. (PDF)

S3 File TREND statement checklist. (PDF)
