# Supplementary material for: Sunscreen use optimized by two consecutive applications
Source: PLoS One. 2018 Mar 28;13(3):e0193916. doi: 10.1371/journal.pone.0193916 (PMC5874020; doi:10.1371/journal.pone.0193916)
Supplement: S1 File — (DOCX) [file pone.0193916.s001.docx]

**S1 File.**

**Log files of the statistical analyses from IBM SPSS Statistics**

**Trial: Optimized sunscreen use by two consecutive sunscreen applications**

**Analysis concerning missed areas**

| **Test Statistics^a,b^** | |
| --- | --- |
|  | Missed area, 2 - Missed area, 1 |
| Z | -4,302^c^ |
| Asymp. Sig. (2-tailed) | ,000 |
| Exact Sig. (2-tailed) | ,000 |
| Exact Sig. (1-tailed) | ,000 |
| Point Probability | ,000 |
| a. Body region = Face | |
| b. Wilcoxon Signed Ranks Test | |
| c. Based on positive ranks. | |

| **Test Statistics^a,b^** | |
| --- | --- |
|  | Missed area, 2 - Missed area, 1 |
| Z | -3,319^c^ |
| Asymp. Sig. (2-tailed) | ,001 |
| Exact Sig. (2-tailed) | ,000 |
| Exact Sig. (1-tailed) | ,000 |
| Point Probability | ,000 |
| a. Body region = Ears | |
| b. Wilcoxon Signed Ranks Test | |
| c. Based on positive ranks. | |

| **Test Statistics^a,b^** | |
| --- | --- |
|  | Missed area, 2 - Missed area, 1 |
| Z | -4,108^c^ |
| Asymp. Sig. (2-tailed) | ,000 |
| Exact Sig. (2-tailed) | ,000 |
| Exact Sig. (1-tailed) | ,000 |
| Point Probability | ,000 |
| a. Body region = Neck, front | |
| b. Wilcoxon Signed Ranks Test | |
| c. Based on positive ranks. | |

| **Test Statistics^a,b^** | |
| --- | --- |
|  | Missed area, 2 - Missed area, 1 |
| Z | -3,636^c^ |
| Asymp. Sig. (2-tailed) | ,000 |
| Exact Sig. (2-tailed) | ,000 |
| Exact Sig. (1-tailed) | ,000 |
| Point Probability | ,000 |
| a. Body region = Neck, back | |
| b. Wilcoxon Signed Ranks Test | |
| c. Based on positive ranks. | |

| **Test Statistics^a,b^** | |
| --- | --- |
|  | Missed area, 2 - Missed area, 1 |
| Z | -4,618^c^ |
| Asymp. Sig. (2-tailed) | ,000 |
| Exact Sig. (2-tailed) | ,000 |
| Exact Sig. (1-tailed) | ,000 |
| Point Probability | ,000 |
| a. Body region = Trunk, front | |
| b. Wilcoxon Signed Ranks Test | |
| c. Based on positive ranks. | |

| **Test Statistics^a,b^** | |
| --- | --- |
|  | Missed area, 2 - Missed area, 1 |
| Z | -4,597^c^ |
| Asymp. Sig. (2-tailed) | ,000 |
| Exact Sig. (2-tailed) | ,000 |
| Exact Sig. (1-tailed) | ,000 |
| Point Probability | ,000 |
| a. Body region = Trunk, back | |
| b. Wilcoxon Signed Ranks Test | |
| c. Based on positive ranks. | |

| **Test Statistics^a,b^** | |
| --- | --- |
|  | Missed area, 2 - Missed area, 1 |
| Z | -4,432^c^ |
| Asymp. Sig. (2-tailed) | ,000 |
| Exact Sig. (2-tailed) | ,000 |
| Exact Sig. (1-tailed) | ,000 |
| Point Probability | ,000 |
| a. Body region = Arms | |
| b. Wilcoxon Signed Ranks Test | |
| c. Based on positive ranks. | |

| **Test Statistics^a,b^** | |
| --- | --- |
|  | Missed area, 2 - Missed area, 1 |
| Z | -3,857^c^ |
| Asymp. Sig. (2-tailed) | ,000 |
| Exact Sig. (2-tailed) | ,000 |
| Exact Sig. (1-tailed) | ,000 |
| Point Probability | ,000 |
| a. Body region = Hands, back | |
| b. Wilcoxon Signed Ranks Test | |
| c. Based on positive ranks. | |

| **Test Statistics^a,b^** | |
| --- | --- |
|  | Missed area, 2 - Missed area, 1 |
| Z | -4,782^c^ |
| Asymp. Sig. (2-tailed) | ,000 |
| Exact Sig. (2-tailed) | ,000 |
| Exact Sig. (1-tailed) | ,000 |
| Point Probability | ,000 |
| a. Body region = Thighs | |
| b. Wilcoxon Signed Ranks Test | |
| c. Based on positive ranks. | |

| **Test Statistics^a,b^** | |
| --- | --- |
|  | Missed area, 2 - Missed area, 1 |
| Z | -4,012^c^ |
| Asymp. Sig. (2-tailed) | ,000 |
| Exact Sig. (2-tailed) | ,000 |
| Exact Sig. (1-tailed) | ,000 |
| Point Probability | ,000 |
| a. Body region = Lower leg | |
| b. Wilcoxon Signed Ranks Test | |
| c. Based on positive ranks. | |

| **Test Statistics^a,b^** | |
| --- | --- |
|  | Missed area, 2 - Missed area, 1 |
| Z | -4,143^c^ |
| Asymp. Sig. (2-tailed) | ,000 |
| Exact Sig. (2-tailed) | ,000 |
| Exact Sig. (1-tailed) | ,000 |
| Point Probability | ,000 |
| a. Body region = Instep | |
| b. Wilcoxon Signed Ranks Test | |
| c. Based on positive ranks. | |

Total missed area

| **Test Statistics^a^** | |
| --- | --- |
|  | Missed area, 2 - Missed area, 1 |
| Z | -4,860^b^ |
| Asymp. Sig. (2-tailed) | ,000 |
| Exact Sig. (2-tailed) | ,000 |
| Exact Sig. (1-tailed) | ,000 |
| Point Probability | ,000 |
| a. Wilcoxon Signed Ranks Test | |
| b. Based on positive ranks. | |

| **Test Statistics^a,b^** | | |
| --- | --- | --- |
|  | Missed area, 1 | Missed area, 2 |
| Mann-Whitney U | 102,500 | 113,500 |
| Wilcoxon W | 222,500 | 249,500 |
| Z | -,692 | -,257 |
| Asymp. Sig. (2-tailed) | ,489 | ,797 |
| Exact Sig. [2*(1-tailed Sig.)] | ,495^c^ | ,800^c^ |
| Exact Sig. (2-tailed) | ,501 | ,808 |
| Exact Sig. (1-tailed) | ,251 | ,404 |
| Point Probability | ,007 | ,008 |
| a. Body region = Face | | |
| b. Grouping Variable: Sex | | |
| c. Not corrected for ties. | | |

| **Test Statistics^a,b^** | | |
| --- | --- | --- |
|  | Missed area, 1 | Missed area, 2 |
| Mann-Whitney U | 35,000 | 31,000 |
| Wilcoxon W | 171,000 | 167,000 |
| Z | -3,208 | -3,203 |
| Asymp. Sig. (2-tailed) | ,001 | ,001 |
| Exact Sig. [2*(1-tailed Sig.)] | ,001^c^ | ,001^c^ |
| Exact Sig. (2-tailed) | ,001 | ,001 |
| Exact Sig. (1-tailed) | ,000 | ,000 |
| Point Probability | ,000 | ,000 |
| a. Body region = Ears | | |
| b. Grouping Variable: Sex | | |
| c. Not corrected for ties. | | |

| **Test Statistics^a,b^** | | |
| --- | --- | --- |
|  | Missed area, 1 | Missed area, 2 |
| Mann-Whitney U | 106,500 | 108,500 |
| Wilcoxon W | 242,500 | 228,500 |
| Z | -,534 | -,167 |
| Asymp. Sig. (2-tailed) | ,593 | ,867 |
| Exact Sig. [2*(1-tailed Sig.)] | ,599^c^ | ,870^c^ |
| Exact Sig. (2-tailed) | ,605 | ,876 |
| Exact Sig. (1-tailed) | ,302 | ,438 |
| Point Probability | ,007 | ,008 |
| a. Body region = Neck, Front | | |
| b. Grouping Variable: Sex | | |
| c. Not corrected for ties. | | |

| **Test Statistics^a,b^** | | |
| --- | --- | --- |
|  | Missed area, 1 | Missed area, 2 |
| Mann-Whitney U | 87,000 | 101,500 |
| Wilcoxon W | 223,000 | 237,500 |
| Z | -1,050 | -,448 |
| Asymp. Sig. (2-tailed) | ,294 | ,654 |
| Exact Sig. [2*(1-tailed Sig.)] | ,313^c^ | ,667^c^ |
| Exact Sig. (2-tailed) | ,303 | ,666 |
| Exact Sig. (1-tailed) | ,152 | ,333 |
| Point Probability | ,005 | ,008 |
| a. Body region = Neck, back | | |
| b. Grouping Variable: Sex | | |
| c. Not corrected for ties. | | |

| **Test Statistics^a,b^** | | |
| --- | --- | --- |
|  | Missed area, 1 | Missed area, 2 |
| Mann-Whitney U | 115,000 | 96,000 |
| Wilcoxon W | 251,000 | 232,000 |
| Z | -,198 | -,949 |
| Asymp. Sig. (2-tailed) | ,843 | ,343 |
| Exact Sig. [2*(1-tailed Sig.)] | ,861^c^ | ,358^c^ |
| Exact Sig. (2-tailed) | ,861 | ,353 |
| Exact Sig. (1-tailed) | ,430 | ,176 |
| Point Probability | ,015 | ,005 |
| a. Body region = Trunk, front | | |
| b. Grouping Variable: Sex | | |
| c. Not corrected for ties. | | |

| **Test Statistics^a,b^** | | |
| --- | --- | --- |
|  | Missed area, 1 | Missed area, 2 |
| Mann-Whitney U | 110,000 | 108,500 |
| Wilcoxon W | 246,000 | 228,500 |
| Z | -,395 | -,455 |
| Asymp. Sig. (2-tailed) | ,693 | ,649 |
| Exact Sig. [2*(1-tailed Sig.)] | ,711^c^ | ,654^c^ |
| Exact Sig. (2-tailed) | ,711 | ,662 |
| Exact Sig. (1-tailed) | ,356 | ,331 |
| Point Probability | ,014 | ,008 |
| a. Body region = Trunk, back | | |
| b. Grouping Variable: Sex | | |
| c. Not corrected for ties. | | |

| **Test Statistics^a,b^** | | |
| --- | --- | --- |
|  | Missed area, 1 | Missed area, 2 |
| Mann-Whitney U | 112,000 | 107,000 |
| Wilcoxon W | 248,000 | 243,000 |
| Z | -,316 | -,514 |
| Asymp. Sig. (2-tailed) | ,752 | ,607 |
| Exact Sig. [2*(1-tailed Sig.)] | ,770^c^ | ,626^c^ |
| Exact Sig. (2-tailed) | ,770 | ,626 |
| Exact Sig. (1-tailed) | ,385 | ,313 |
| Point Probability | ,015 | ,014 |
| a. Body region = Arms | | |
| b. Grouping Variable: Sex | | |
| c. Not corrected for ties. | | |

| **Test Statistics^a,b^** | | |
| --- | --- | --- |
|  | Missed area, 1 | Missed area, 2 |
| Mann-Whitney U | 97,000 | 84,000 |
| Wilcoxon W | 202,000 | 175,000 |
| Z | -,046 | -,902 |
| Asymp. Sig. (2-tailed) | ,963 | ,367 |
| Exact Sig. [2*(1-tailed Sig.)] | ,982^c^ | ,398^c^ |
| Exact Sig. (2-tailed) | ,972 | ,384 |
| Exact Sig. (1-tailed) | ,486 | ,192 |
| Point Probability | ,008 | ,012 |
| a. Body region = Hands, back | | |
| b. Grouping Variable: Sex | | |
| c. Not corrected for ties. | | |

| **Test Statistics^a,b^** | | |
| --- | --- | --- |
|  | Missed area, 1 | Missed area, 2 |
| Mann-Whitney U | 110,500 | 94,000 |
| Wilcoxon W | 230,500 | 214,000 |
| Z | -,376 | -1,028 |
| Asymp. Sig. (2-tailed) | ,707 | ,304 |
| Exact Sig. [2*(1-tailed Sig.)] | ,711^c^ | ,318^c^ |
| Exact Sig. (2-tailed) | ,719 | ,318 |
| Exact Sig. (1-tailed) | ,360 | ,159 |
| Point Probability | ,008 | ,009 |
| a. Body region = Thighs | | |
| b. Grouping Variable: Sex | | |
| c. Not corrected for ties. | | |

| **Test Statistics^a,b^** | | |
| --- | --- | --- |
|  | Missed area, 1 | Missed area, 2 |
| Mann-Whitney U | 80,000 | 95,500 |
| Wilcoxon W | 200,000 | 215,500 |
| Z | -1,584 | -,973 |
| Asymp. Sig. (2-tailed) | ,113 | ,331 |
| Exact Sig. [2*(1-tailed Sig.)] | ,119^c^ | ,338^c^ |
| Exact Sig. (2-tailed) | ,117 | ,340 |
| Exact Sig. (1-tailed) | ,058 | ,170 |
| Point Probability | ,002 | ,005 |
| a. Body region = Lower leg | | |
| b. Grouping Variable: Sex | | |
| c. Not corrected for ties. | | |

| **Test Statistics^a,b^** | | |
| --- | --- | --- |
|  | Missed area, 1 | Missed area, 2 |
| Mann-Whitney U | 99,000 | 112,000 |
| Wilcoxon W | 219,000 | 217,000 |
| Z | -,840 | ,000 |
| Asymp. Sig. (2-tailed) | ,401 | 1,000 |
| Exact Sig. [2*(1-tailed Sig.)] | ,423^c^ | 1,000^c^ |
| Exact Sig. (2-tailed) | ,417 | 1,000 |
| Exact Sig. (1-tailed) | ,208 | ,508 |
| Point Probability | ,011 | ,016 |
| a. Body region = Instep | | |
| b. Grouping Variable: Sex | | |
| c. Not corrected for ties. | | |

**Analysis concerning quantities of sunscreen**

| **Test Statistics^a,b^** | |
| --- | --- |
|  | Quantity of sunscreen, 2 - Quantity of sunscreen, 1 |
| Z | -1,960^c^ |
| Asymp. Sig. (2-tailed) | ,050 |
| Exact Sig. (2-tailed) | 0,049613 |
| Exact Sig. (1-tailed) | ,025 |
| Point Probability | ,001 |
| a. Skin site = Shoulder | |
| b. Wilcoxon Signed Ranks Test | |
| c. Based on negative ranks. | |
| **Test Statistics^a,b^** | |
|  | Quantity of sunscreen, 2 - Quantity of sunscreen, 1 |
| Z | -2,733^c^ |
| Asymp. Sig. (2-tailed) | ,006 |
| Exact Sig. (2-tailed) | ,005 |
| Exact Sig. (1-tailed) | ,003 |
| Point Probability | ,000 |
| a. Skin site = Forehead | |
| b. Wilcoxon Signed Ranks Test | |
| c. Based on negative ranks. | |

| **Test Statistics^a,b^** | |
| --- | --- |
|  | Quantity of sunscreen, 2 - Quantity of sunscreen, 1 |
| Z | -3,783^c^ |
| Asymp. Sig. (2-tailed) | ,000 |
| Exact Sig. (2-tailed) | ,000 |
| Exact Sig. (1-tailed) | ,000 |
| Point Probability | ,000 |
| a. Skin site = Chest | |
| b. Wilcoxon Signed Ranks Test | |
| c. Based on negative ranks. | |

| **Test Statistics^a,b^** | |
| --- | --- |
|  | Quantity of sunscreen, 2 - Quantity of sunscreen, 1 |
| Z | -2,962^c^ |
| Asymp. Sig. (2-tailed) | ,003 |
| Exact Sig. (2-tailed) | ,002 |
| Exact Sig. (1-tailed) | ,001 |
| Point Probability | ,000 |
| a. Skin site = Belly | |
| b. Wilcoxon Signed Ranks Test | |
| c. Based on negative ranks. | |

| **Test Statistics^a,b^** | |
| --- | --- |
|  | Quantity of sunscreen, 2 - Quantity of sunscreen, 1 |
| Z | -2,822^c^ |
| Asymp. Sig. (2-tailed) | ,005 |
| Exact Sig. (2-tailed) | ,004 |
| Exact Sig. (1-tailed) | ,002 |
| Point Probability | ,000 |
| a. Skin site = Lower leg | |
| b. Wilcoxon Signed Ranks Test | |
| c. Based on negative ranks. | |

| **Test Statistics^a,b^** | |
| --- | --- |
|  | Quantity of sunscreen, 2 - Quantity of sunscreen, 1 |
| Z | -3,445^c^ |
| Asymp. Sig. (2-tailed) | ,001 |
| Exact Sig. (2-tailed) | ,000 |
| Exact Sig. (1-tailed) | ,000 |
| Point Probability | ,000 |
| a. Skin site = Thigh | |
| b. Wilcoxon Signed Ranks Test | |
| c. Based on negative ranks. | |

| **Test Statistics^a,b^** | |
| --- | --- |
|  | Quantity of sunscreen, 2 - Quantity of sunscreen, 1 |
| Z | -1,322^c^ |
| Asymp. Sig. (2-tailed) | ,186 |
| Exact Sig. (2-tailed) | ,194 |
| Exact Sig. (1-tailed) | ,097 |
| Point Probability | ,004 |
| a. Skin site = Upper back | |
| b. Wilcoxon Signed Ranks Test | |
| c. Based on negative ranks. | |

| **Test Statistics^a,b^** | | |
| --- | --- | --- |
|  | Quantity of sunscreen, 1 | Quantity of sunscreen, 2 |
| Mann-Whitney U | 75,000 | 111,500 |
| Wilcoxon W | 211,000 | 247,500 |
| Z | -1,779 | -,336 |
| Asymp. Sig. (2-tailed) | ,075 | ,737 |
| Exact Sig. [2*(1-tailed Sig.)] | ,078^c^ | ,740^c^ |
| Exact Sig. (2-tailed) | ,078 | ,748 |
| Exact Sig. (1-tailed) | ,039 | ,374 |
| Point Probability | ,003 | ,008 |
| a. Skin site = Shoulder | | |
| b. Grouping Variable: Sex | | |
| c. Not corrected for ties. | | |

| **Test Statistics^a,b^** | | |
| --- | --- | --- |
|  | Quantity of sunscreen, 1 | Quantity of sunscreen, 2 |
| Mann-Whitney U | 109,500 | 110,000 |
| Wilcoxon W | 245,500 | 246,000 |
| Z | -,104 | -,083 |
| Asymp. Sig. (2-tailed) | ,917 | ,934 |
| Exact Sig. [2*(1-tailed Sig.)] | ,918^c^ | ,951^c^ |
| Exact Sig. (2-tailed) | ,926 | ,951 |
| Exact Sig. (1-tailed) | ,463 | ,476 |
| Point Probability | ,008 | ,016 |
| a. Skin site = Forehead | | |
| b. Grouping Variable: Sex | | |
| c. Not corrected for ties. | | |

| **Test Statistics^a,b^** | | |
| --- | --- | --- |
|  | Quantity of sunscreen, 1 | Quantity of sunscreen, 2 |
| Mann-Whitney U | 112,000 | 111,500 |
| Wilcoxon W | 232,000 | 231,500 |
| Z | -,316 | -,336 |
| Asymp. Sig. (2-tailed) | ,752 | ,737 |
| Exact Sig. [2*(1-tailed Sig.)] | ,770^c^ | ,740^c^ |
| Exact Sig. (2-tailed) | ,770 | ,748 |
| Exact Sig. (1-tailed) | ,385 | ,374 |
| Point Probability | ,015 | ,007 |
| a. Skin site = Chest | | |
| b. Grouping Variable: Sex | | |
| c. Not corrected for ties. | | |

| **Test Statistics^a,b^** | | |
| --- | --- | --- |
|  | Quantity of sunscreen, 1 | Quantity of sunscreen, 2 |
| Mann-Whitney U | 109,000 | 111,000 |
| Wilcoxon W | 245,000 | 247,000 |
| Z | -,436 | -,042 |
| Asymp. Sig. (2-tailed) | ,663 | ,967 |
| Exact Sig. [2*(1-tailed Sig.)] | ,682^c^ | ,984^c^ |
| Exact Sig. (2-tailed) | ,674 | ,976 |
| Exact Sig. (1-tailed) | ,337 | ,488 |
| Point Probability | ,007 | ,008 |
| a. Skin site = Belly | | |
| b. Grouping Variable: Sex | | |
| c. Not corrected for ties. | | |

| **Test Statistics^a,b^** | | |
| --- | --- | --- |
|  | Quantity of sunscreen, 1 | Quantity of sunscreen, 2 |
| Mann-Whitney U | 94,000 | 103,000 |
| Wilcoxon W | 230,000 | 239,000 |
| Z | -1,028 | -,672 |
| Asymp. Sig. (2-tailed) | ,304 | ,501 |
| Exact Sig. [2*(1-tailed Sig.)] | ,318^c^ | ,520^c^ |
| Exact Sig. (2-tailed) | ,313 | ,513 |
| Exact Sig. (1-tailed) | ,157 | ,257 |
| Point Probability | ,005 | ,006 |
| a. Skin site = Lower leg | | |
| b. Grouping Variable: Sex | | |
| c. Not corrected for ties. | | |

| **Test Statistics^a,b^** | | |
| --- | --- | --- |
|  | Quantity of sunscreen, 1 | Quantity of sunscreen, 2 |
| Mann-Whitney U | 107,000 | 97,000 |
| Wilcoxon W | 243,000 | 233,000 |
| Z | -,514 | -,909 |
| Asymp. Sig. (2-tailed) | ,607 | ,363 |
| Exact Sig. [2*(1-tailed Sig.)] | ,626^c^ | ,379^c^ |
| Exact Sig. (2-tailed) | ,619 | ,374 |
| Exact Sig. (1-tailed) | ,309 | ,187 |
| Point Probability | ,007 | ,005 |
| a. Skin site = Thigh | | |
| b. Grouping Variable: Sex | | |
| c. Not corrected for ties. | | |

| **Test Statistics^a,b^** | | |
| --- | --- | --- |
|  | Quantity of sunscreen, 1 | Quantity of sunscreen, 2 |
| Mann-Whitney U | 74,000 | 97,500 |
| Wilcoxon W | 210,000 | 233,500 |
| Z | -1,830 | -,893 |
| Asymp. Sig. (2-tailed) | ,067 | ,372 |
| Exact Sig. [2*(1-tailed Sig.)] | ,072^c^ | ,379^c^ |
| Exact Sig. (2-tailed) | ,068 | ,382 |
| Exact Sig. (1-tailed) | ,034 | ,191 |
| Point Probability | ,001 | ,005 |
| a. Skin site = Upper back | | |
| b. Grouping Variable: Sex | | |
| c. Not corrected for ties. | | |

**Validation of picture analysis**

| **Correlations** | | | | |
| --- | --- | --- | --- | --- |
|  | | | Quantity of sunscreen, picture | Quantity of sunscreen, weigh |
| Spearman's rho | Quantity of sunscreen, picture | Correlation Coefficient | 1,000 | ,792^**^ |
|  |  | Sig. (2-tailed) | . | ,000 |
|  |  | N | 62 | 61 |
|  | Quantity of sunscreen, weigh | Correlation Coefficient | ,792^**^ | 1,000 |
|  |  | Sig. (2-tailed) | ,000 | . |
|  |  | N | 61 | 61 |
| **. Correlation is significant at the 0.01 level (2-tailed). | | | | |
