# Supplementary material for: Sunscreen use optimized by two consecutive applications
Source: PLoS One. 2018 Mar 28;13(3):e0193916. doi: 10.1371/journal.pone.0193916 (PMC5874020; doi:10.1371/journal.pone.0193916)
Supplement: S2 File — (PDF) [file pone.0193916.s002.pdf]

S2 File.
Clinical trial protocol

Trial: Optimized sunscreen use by two consecutive sunscreen applications

Trial registration clinicaltrials.gov identifier: NCT03033654

Sponsor: Professor Hans Christian Wulf, MD, DMSc, PharmD

Department of Dermatology, Bispebjerg Hospital, Bispebjerg Bakke 23, 2400 København NV

Investigator: Ida Heerfordt, MD

Department of Dermatology, Bispebjerg Hospital, Bispebjerg Bakke 23, 2400 København NV

E-mail: ida.marie.heerfordt@regionh.dk

Sub-investigator: Linnea Tornes, MD

Department of Dermatology, Bispebjerg Hospital, Bispebjerg Bakke 23, 2400 København NV

Time schedule:

Inclusion start: November 2014

Trial period: From December 2014 to December 2015

Follow-up: None

HC Wulf is responsible for conducting the trial in accordance with the guidelines in the protocol.

Summary

Title: Two consecutive sunscreen applications

Sponsor: Professor Hans Christian Wulf, MD, DMSc, PharmD

Importance: Sunscreen users are often inadequately protected and become sunburned.

Objectives: To investigate how much two consecutive applications of sunscreen before sun exposure increase the quantity of sunscreen applied and decrease the skin area left without sunscreen compared to a single application.

Design: Intraindividual intervention study to be conducted at Bispebjerg Hospital, Copenhagen, Denmark.

Setting: Volunteers wearing swimwear will apply sunscreen twice in a laboratory environment and have pictures taken in black light before and after. We will conduct a standard curve establishing a link between skin picture darkness and quantity of sunscreen applied.

Sunscreen: During the experiment, Actinica® (Galderma) sunscreen is used. It has a sun protection factor of 50+.

Participants: Two groups: 15 women and 15 men.

Inclusion criteria: Healthy, older than 18 years of age, and of European descent.

Exclusion criteria: Suffering from a skin disease, allergic sunscreen content, pregnant or breastfeeding.

Side effects and safety assessment: Our subjects are not exposed to any risks. The black light cabinet emits UVA1 in completely harmless doses when used for picture taking only.

Financial conditions: The trial has been initiated by Professor Hans Christian Wulf. The costs of the experiment are covered by internal funds from Department of Dermatology, Bispebjerg Hospital. The study staff has no financial interests in the experiment. Subjects receive a fee of 400 DKK for participation in the trial. The fee is paid by Bispebjerg Hospital.

Publication: The results are scheduled to be published in an international journal. The results will be published regardless of whether the outcome of the study can be interpreted as positive, negative or inconclusive.

1. Background

Ultraviolet (UV) radiation is the primary cause of skin cancer. The World Health Organization (WHO) has presented some simple guidelines for protection against harmful UV rays. It is recommended to avoid the sun in the middle of the day, stay in the shade, wear protective clothes, avoid solarium and use sunscreen.[1, 2] Use of a sunscreen with a sun protection factor (SPF) of at least 15 is recommended and it should be used 20 minutes before sun exposure, every second hour and after swimming or physical exercise.[3]

The amount of sunscreen used when testing the SPF is 2 mg/cm^2. It has been shown that people typically only use 20-50% of this amount. Consequently, the actual protection will be significantly lower than the labeled SPF. [4]

Missed areas are accessible skin areas left without sunscreen after application. Studies have shown that people usually do not succeed in covering the entire body in sunscreen.[5, 6] Some studies have shown that you can increase the protected body area by applying sunscreen twice.[7, 8]

2. Aim

To investigate how much two consecutive applications of sunscreen before sun exposure will increase the quantity of sunscreen applied and decrease the skin area left without sunscreen compared to a single application.

3. Study plan and design

3.1 Outcomes

Main outcome:

- Estimate how much two consecutive applications of sunscreen before sun exposure will increase the quantity of sunscreen applied and decrease the skin area left without sunscreen compared to a single application.

Other outcomes:

- Estimate the percentage of skin area left without sunscreen in different body locations (face, ears, neck, trunk, arms, hands, thighs, lower leg, and instep) and in total. Both after one and two applications.
- Establish a standard curve linking skin photodarkness and sunscreen application thickness in mg/cm^2.
- Estimate sunscreen application thickness at 7 different skin sites (forehead, chest, shoulder, upper back, belly, thigh and lower leg). After one and two applications.
- Check if there is a difference between women’s and men’s application patterns.

- Estimate how long time the subjects spend on applying sunscreen.
- Calculate tolerable UVR doses depending on sunscreen use.

3.2 Study design

Intraindividual intervention study.

3.3 Randomization and blinding:

There is no randomization or blinding.

3.4 Equipment

Black light cabin: UV radiation in the cabinet is absorbed by applied sunscreen. Type: TLD 18 W/08. Producent: Philips, Amsterdam, The Netherlands.

Camera: A Canon EOS 450D is used to obtain photographs. The settings are as follows; exposure time: 1/15 seconds, ISO speed: 1600, f-stop: f/5.6, focal length: 35mm.

Weight: For accurate measurement of the amount of sunscreen applied the following weight is used: Model: AB204. Producent: Mettler-Toledo, Leicester, United Kingdom.

Sunscreen: The sunscreen used for testing is Actinica® (SPF 50+). Galderma, Switzerland.

3.5 Study procedure

The investigation of a participant is conducted on a single study day after the following procedure:

(1) Subjects report their height and weight for calculation of body surface area.

(2) Women wear a bikini. Men wear swimming trunks.

(3) The dose response relationship between sunscreen application thickness and picture darkness is investigated in all subjects. Six equal squares (30cm^2 large each) are marked on the subjects' lower backs. Sunscreen is applied with increasing amount in each square; 0 mg/cm^2; 0.25 mg/ cm^2; 0.5 mg/cm^2; 1.0 mg/cm^2; 1.5 mg/cm^2; and 2.0 mg/cm^2. Pictures are taken in black light before and after application with a fixed distance of 43 cm and 2 m. Fluorescence photos will be analyzed using an imaging-editing program (GIMP 2.8.14 http://www.gimp.org/).

(4) The subjects are asked to apply sunscreen the same way they would normally do on a sunny day at the beach in Denmark disregarding the squares on their lower bag. No other information or advice is given.

Time spent on sunscreen application is noted. Then pictures are taken in black light of the whole body with fixed distances 43 cm and 2 meters.

(5) After 20 minutes step 4 is repeated. The sunscreen container is weighed before and after each application.

Based on the photos we hope to estimate the areas not covered by sunscreen and the thickness of sunscreen applied.

4. Participants

Two groups: 15 women and 15 men.

Inclusion criteria: Healthy, older than 18 years of age, and of European descent.

Exclusion criteria: Suffering from a skin disease, allergic to sunscreen content, pregnant or breastfeeding.

4.1 Exit of the experiment

Participants will exit the test in the following circumstances: According to the subject's own request.

Subjects leaving the study are replaced by other subjects.

4.2 Payment

Subjects receive a fee of 400 DKK for participation in the trial.

5. Impact assessment

The level of protection at selected skin sites as well as the percentage of skin area left without sunscreen (missed areas) are determined in different body regions and in total.

6. Safety assessment

No adverse effects are expected. Allergy to sunscreen or photoallergy may occur, but is rare. Black light contains UVA 1. Maximum intensity of UVA1 is 0.1 mW/cm^2 which the patient is exposed to for 1 minute. This corresponds to 6 mJ/cm^2 in total, which is completely harmless.

7. Statistics

7.1 Statistical methods

The statistical analysis is performed with IBM SPSS statistics version 22 (IBM, Armonk, NY, USA). Data will be described with descriptive statistics. P-values less than 0.05 are considered significant.

7.2 Sample size

Sample size requirement was based on a study comparing quantity of sunscreen after single and double application.[8] Quantity of sunscreen was estimated to be 1.27 mg/cm^2 after single application with a standard deviation (SD) of 0.47 mg/cm^2 and after double application 2.01mg/cm^2 with a SD of 0.66 mg/cm^2. To allow detection of difference in quantity after single and double application with a power of 80% and a significance level of 5% we needed 10 participants. As men and women might apply sunscreen differently we chose to include at least 15 men and 15 women.[9, 10]

8. Data, quality control and security

8.1 Data

The sponsor allows direct access to data and documents for monitoring, auditing and inspection from the Danish Health Authorities. The project has been reported to and approved by the Danish Data Protection Agency.

8.2 Quality control and quality assurance procedures

The experiment is performed according to current guidelines from the Danish Health Authorities. The project is submitted to the Scientific Ethics Committee. A list of included subjects' names, telephone numbers and dates of birth will be stored. The test subjects are informed that data will be stored and analyzed according to current Danish legislation.

9. Handling and archiving of data

Data is stored at Department of Dermatology, Bispebjerg Hospital for 5 years.

10. Ethics

10.1 Ethical statement

The protocol will be submitted to the Scientific Ethics Committee for Capital Region. The experiment is considered very safe and is not considered to contain ethical problems. Photoallergy can occur but is very rare. Black light emits UVA1 rays, which in the given doses are completely harmless.

10.2 Information from study staff to subjects

The investigators are responsible for informing the participants about the purpose of the experiment, structure and possible risks. The oral information is given on a pre-agreed time prior to inclusion in the trial. The investigators ensure that the subject does understand the information before written informed consent is obtained. The subjects are informed that they can withdraw their informed consent at any time. A copy of the written consent is handed to the subject and the original is stored by the investigator in the patient record. Informed consent to publish the photos is also required by the subjects. This is stored with the informed consent statement.

11. Financing and insurance

11.1 Financing

The trial has been initiated by Professor Hans Christian Wulf. The costs of the experiment are covered by internal funds from Department of Dermatology, Bispebjerg Hospital. The study staff has no financial interests in the experiment.

Subjects receive a fee of 400 DKK for participation in the trial. The fee is paid by Bispebjerg Hospital and is taxed normally.

11.2 Insurance

With regard to insurance and compensation claims, Danish legislation is followed. All treatments are performed at the Department of Dermatology, Bispebjerg Hospital, and the subjects are covered by the hospital's patient insurance.

12. Publication

The results are scheduled to be published in an international journal. Furthermore, the results will be presented on international conferences.

The results will be published regardless of whether the outcome of the study can be interpreted as positive, negative or inconclusive.

Reference List

1. Green,A. *et al.* Daily sunscreen application and betacarotene supplementation in prevention of basal-cell and squamous-cell carcinomas of the skin: a randomised controlled trial. *Lancet* **354**, 723-729 (1999).

2. Lautenschlager,S., Wulf,H.C., & Pittelkow,M.R. Photoprotection. *Lancet* **370**, 528-537 (2007).

3. Petersen,B. & Wulf,H.C. Application of sunscreen--theory and reality. *Photodermatol. Photoimmunol. Photomed.* **30**, 96-101 (2014).

4. Bech-Thomsen,N. & Wulf,H.C. Sunbathers' application of sunscreen is probably inadequate to obtain the sun protection factor assigned to the preparation. *Photodermatol. Photoimmunol. Photomed.* **9**, 242-244 (1992).

5. Jeanmougin,M., Bouloc,A., & Schmutz,J.L. A new sunscreen application technique to protect more efficiently from ultraviolet radiation. *Photodermatol. Photoimmunol. Photomed.* **30**, 323-331 (2014).

6. Lademann,J. *et al.* Sunscreen application at the beach. *J. Cosmet. Dermatol.* **3**, 62-68 (2004).

7. Odio,M.R. *et al.* Comparative efficacy of sunscreen reapplication regimens in children exposed to ambient sunlight. *Photodermatol. Photoimmunol. Photomed.* **10**, 118-125 (1994).

8. Teramura,T. *et al.* Relationship between sun-protection factor and application thickness in high-performance sunscreen: double application of sunscreen is recommended. *Clin. Exp. Dermatol.* **37**, 904-908 (2012).

9. Petersen,B., Datta,P., Philipsen,P.A., & Wulf,H.C. Sunscreen use and failures--on site observations on a sun-holiday. *Photochem. Photobiol. Sci.* **12**, 190-196 (2013).

10. Gaughan,M.D. & Padilla,R.S. Use of a topical fluorescent dye to evaluate effectiveness of sunscreen application. *Arch. Dermatol.* **134**, 515-517 (1998).
